# Pathogen-Specific Temporal Coupling Between Outpatient Influenza-like Illness and Hospitalized Severe Acute Respiratory Infection Positivity in China: A National Sentinel Surveillance Time-Series Study

**DOI:** 10.3390/pathogens15101081

**Published:** 2026-10-14

**Authors:** Qiong Chen, Shanshan Tong, Jun Gao, Sufang Xu

**Affiliations:** 1 Department of Medical Laboratory Center, Anhui No. 2 Provincial People’s Hospital, Hefei 230041, China; cqsey11@163.com (Q.C.);; 2 Faculty of Chinese Medicine, Macau University of Science and Technology, Avenida Wai Long, Taipa, Macao 999078, China

**Keywords:** respiratory pathogen surveillance, influenza-like illness, severe acute respiratory infection, distributed lag model, time-series analysis, cross-correlation

## Abstract

Outpatient influenza-like illness (ILI) and hospitalized severe acute respiratory infection (SARI) surveillance measure respiratory-pathogen activity in distinct clinical settings. We analyzed 95 epidemiological weeks (2024-W46 to 2026-W36) of national China CDC positivity data for ten respiratory viruses and *Mycoplasma pneumoniae*. For each pathogen, weekly change in SARI positivity was regressed on ILI changes at lags 1–4, with adjustment for four SARI autoregressive lags and annual seasonality. Inference used Newey–West covariance, joint lag tests, and Benjamini–Hochberg false-discovery-rate correction; reverse-direction, specification, recursive block-bootstrap, prewhitened cross-correlation, rolling-coefficient, and rolling-origin forecast analyses were secondary. Asymptotic forward lag blocks were FDR-significant for ten pathogens, whereas recursive block-bootstrap inference supported six: influenza virus, respiratory syncytial virus, human metapneumovirus, parainfluenza virus, common human coronavirus, and rhinovirus. No augmented model showed FDR-significant evidence of improved forecast error at 1-, 2-, or 4-week horizons; with only 35–38 forecast origins, modest incremental value could not be excluded. National ILI and SARI positivity series showed pathogen-specific temporal coupling, but this 95-week record did not establish fixed lead times or incremental prospective forecasting value in this dataset.

## 1. Introduction

Respiratory infections place recurrent demands on outpatient and hospital services. International standards distinguish influenza-like illness (ILI) surveillance in ambulatory populations from severe acute respiratory infection (SARI) surveillance in hospitalized patients [1,2,3,4]. These systems observe related pathogen activity at different levels of clinical severity and may differ in timing because of care-seeking behavior, age distribution, admission practice, testing policy, and reporting delay [5,6].

Previous cross-setting studies have not identified a uniform temporal ordering. In one integrated health system, outpatient ILI activity preceded inpatient ILI by approximately one week [7]. In Catalonia, primary-care influenza surveillance reached epidemic thresholds before severe hospitalized laboratory-confirmed influenza in most seasons [8]. By contrast, analyses from the Netherlands found that SARI or intensive-care activity could precede primary-care ILI and that the estimated direction and lag varied by season [9,10].

These differences are epidemiologically plausible. Outpatient and inpatient series combine pathogen transmission with age-specific susceptibility, clinical severity, time from symptom onset to presentation or admission, test selection, and reporting procedures. Shared weather, school schedules, vaccination, and healthcare use can also align or displace the two series without a direct progression from ILI to SARI. Consequently, contemporaneous correlation or visually aligned curves cannot establish whether outpatient positivity systematically precedes hospitalized activity [11,12,13,14].

China-based studies have described respiratory-pathogen circulation by age, clinical setting, and phase of the COVID-19 pandemic [15,16,17,18,19,20,21], and pathogen-specific seasonality varies across populations and climates [22,23,24,25,26,27,28]. However, prior work has largely focused on influenza, syndromic counts, or a single clinical setting. It has not compared a common national panel of pathogen-specific ILI and SARI positivity using forward and reverse dynamic models, multiplicity control, finite-sample sensitivity analysis, and time-ordered forecast evaluation. Since November 2024, China CDC has reported such a paired national panel for ten respiratory viruses and *M. pneumoniae* [29]; the available record comprises 95 weeks and fewer than two annual cycles.

We tested whether earlier changes in ILI positivity improved explanation of subsequent changes in SARI positivity beyond SARI history and parsimonious annual adjustment. We prespecified the four-lag block as the primary inferential target, evaluated its sensitivity to stationarity, lag-window, dynamic, seasonal, influence, and bootstrap assumptions, described cross-correlations, and tested whether any in-sample association improved 1-, 2-, or 4-week-ahead forecasts.

## 2. Materials and Methods

### 2.1. Study Design and Reporting

We performed a national ecological time-series study using publicly available weekly summaries from sentinel surveillance. The unit of analysis was the epidemiological week within each pathogen-specific pair of ILI and SARI positivity series. The study was reported in accordance with STROBE principles [30].

### 2.2. Data Source and Surveillance Measures

We extracted China CDC reports covering 2024-W46 through 2026-W36 (11 November 2024 to 6 September 2026). These reports summarized nucleic-acid testing of respiratory specimens from two sentinel populations in mainland China: outpatients or emergency-department patients who met the ILI definition, and hospitalized patients who met the SARI definition. Hong Kong, Macao, and Taiwan were not included [29]. ILI was defined as an acute respiratory infection with measured fever of at least 38 °C and cough, beginning within the previous 10 days. SARI used the same symptom and onset criteria but required hospitalization and allowed either a history of fever or measured fever of at least 38 °C [1,2]. The pathogen panel comprised SARS-CoV-2, influenza virus, respiratory syncytial virus (RSV), adenovirus, human metapneumovirus (hMPV), parainfluenza virus, common human coronavirus other than SARS-CoV-2, bocavirus, rhinovirus, enterovirus, and *M. pneumoniae*.

For each pathogen, the reported measure was the weekly percentage of tested specimens that were positive. The public tables did not report weekly pathogen-specific denominators, sentinel-site composition, or age- and region-stratified values. Weekly positivity estimates therefore could not be weighted by testing volume, and their sampling precision may vary from week to week. We analyzed associations between reported national positivity series, not incidence, hospitalization risk, hospital burden, or progression from outpatient to inpatient care. HAC covariance addresses residual dependence in regression errors; it does not correct measurement error, changing denominators, or shifts in sentinel composition.

### 2.3. Data Extraction and Quality Control

For each weekly value, we retained the week identifier, monitoring date, report title, source URL, and extraction status. Quality checks covered the expected 95-week sequence, duplicate keys, permissible ranges, and completeness of all 22 setting-by-pathogen series. Both ILI and SARI values were available for every one of the 1045 week-pathogen records, yielding 2090 positivity observations. We reconciled the official 2026-W32 values between the long- and wide-format files; no positivity value was missing.

### 2.4. Descriptive Analysis

We calculated means, ranges, and peak weeks. If the maximum occurred in more than one week, all tied weeks were reported. Pearson correlations between undifferenced ILI and SARI levels, together with Fisher-z 95% confidence intervals, were used only for description.

### 2.5. Primary Lagged-Association Model

Before model fitting, we examined each ILI and SARI series using augmented Dickey–Fuller (ADF) and Kwiatkowski–Phillips–Schmidt–Shin (KPSS) tests. Level tests included an intercept and linear trend; tests of first differences included an intercept. The level diagnostics were mixed, whereas the differenced series more consistently satisfied stationarity criteria. First differencing was therefore used to model week-to-week co-movement and to reduce low-frequency trend and shared seasonality. For each pathogen *p* and week index t, with t = 0 corresponding to 2024-W46, we fitted a linear distributed-lag model [31,32]:

ΔS_p_,_t_ = α_p_ + Σ_j=1_^4^ φ_p_,_j_ΔS_p_,_t−j_ + Σ_j=1_^4^ β_p_,_j_ΔI_p_,_t−j_ + γ_p_,s sin(2πt/52.18) + γ_p_,c cos(2πt/52.18) + ε_p_,_t_

Here, ΔS and ΔI denote week-to-week changes in SARI and ILI positivity, expressed in percentage points. Lags 1–4 were prespecified to cover the next four weekly surveillance intervals, a period encompassing short delays between symptom onset, outpatient presentation, deterioration or admission, specimen collection, and reporting. The primary null hypothesis was β_p,1_ = … = β_p,4_ = 0. The reduced model omitted all four ILI terms. In a separate extended model, we added concurrent ΔI_p,t_; lag 0 was not included in the primary lag block. After differencing and allowing for four weeks of history, 90 observations remained (2024-W51 to 2026-W36). Alternative ILI lag windows of 1–2, 1–6, and 1–8 weeks and information-criterion comparisons across maximum lags 1–8 were examined as exploratory sensitivity analyses.

Models were fitted by ordinary least squares. We estimated Bartlett-kernel Newey–West heteroskedasticity- and autocorrelation-consistent covariance matrices using bandwidth 4 and *n*/(*n*−*k*) finite-sample scaling, with the intercept included in k [33]. Two-sided 95% confidence intervals used t(79) quantiles for the primary and reverse models and t(78) quantiles for the extended concurrent model. Joint tests of the four lag parameters used F(4,79). Because these finite-sample reference distributions remain approximate with HAC covariance, we repeated the primary forward block tests using 3999 restricted recursive circular moving-block bootstrap replicates under the reduced model, with block lengths 4 and 8.

The restricted-bootstrap null model omitted the four ILI lags. Residuals from this reduced model were mean-centered and resampled in circular blocks. Each replicate held the observed ILI series fixed, retained the observed initial SARI level and first four SARI differences as initial conditions, recursively generated subsequent SARI differences under the reduced AR(4)-Fourier model, reconstructed the SARI level series, and refitted the full model. The bootstrap *p* value was calculated as (1 + the number of replicate HAC-Wald F statistics at least as large as the observed statistic)/(1 + the number of successful replicates). The resulting inference is conditional on the observed ILI series. The complete implementation is provided in Supplementary File S1.

The confirmatory family comprised the 11 primary forward omnibus tests and was adjusted with the Benjamini–Hochberg procedure [34]. All other tests were designated exploratory. Reverse blocks, cumulative effects, delayed coefficients, concurrent coefficients, specification sensitivities, and forecast comparisons were each adjusted within a separate family defined by a common estimand and analysis. We report effect estimates and 95% confidence intervals together with q values. The cumulative lag effect, Σj = 1…4 βp,j, represents a one-percentage-point increase in ILI positivity in each of the four preceding weeks, not a single one-week increase. For example, the RSV estimate of 1.65 means that four consecutive one-percentage-point weekly ILI increases corresponded to a 1.65-percentage-point-higher modeled weekly change in SARI positivity, conditional on the other covariates.

**Figure 1:**
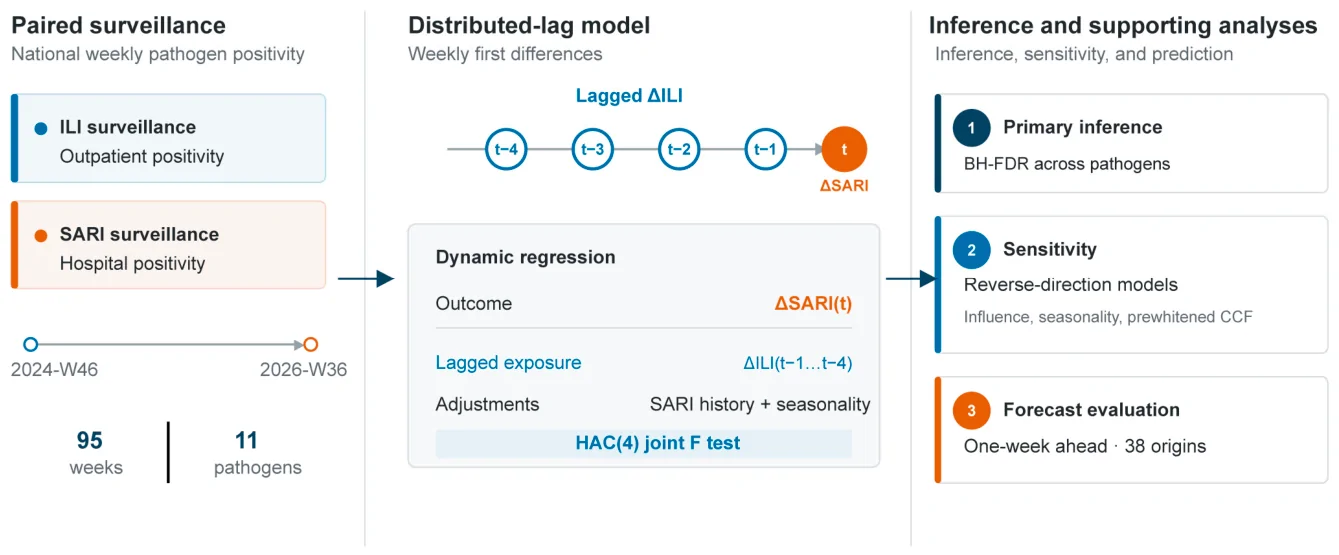
National weekly ILI–SARI surveillance and analysis framework. Weekly pathogen positivity was paired between outpatient ILI and hospitalized SARI surveillance for 95 epidemiological weeks (2024-W46 to 2026-W36) and 11 pathogens. The primary model regressed weekly change in SARI positivity at week t on changes in ILI positivity at lags 1–4, adjusting for four lags of SARI change and one annual Fourier harmonic. Inference used Newey–West heteroskedasticity- and autocorrelation-consistent (HAC) covariance with bandwidth 4, joint F tests, and Benjamini–Hochberg false-discovery-rate (BH-FDR) correction across pathogens. Analyses of concurrent lag 0, reverse direction, influence, temporal stability, prewhitened cross-correlations, and rolling-origin forecasts were secondary. ILI, influenza-like illness; SARI, severe acute respiratory infection; CCF, cross-correlation function. The ellipsis denotes all four lagged ILI terms from *t* − 1 to *t* − 4. Blue identifies the ILI exposure and primary/sensitivity analyses, orange identifies the SARI outcome and forecast evaluation, and the arrows indicate temporal ordering and the direction of the analytical workflow.

We assessed collinearity after residualizing each ILI lag on the SARI lags and Fourier terms. Reported diagnostics include the condition number of the standardized residualized ILI block and the variance-inflation factor (VIF) for each lag. For the primary AR(4) models, residual dependence was evaluated with the Ljung–Box test at lag 8 using model_df = 4, leaving four effective diagnostic degrees of freedom (8 − 4 = 4) [35]. To evaluate dynamic misspecification rather than relying on HAC covariance alone, we also fitted an AR(8) outcome-history model and models with AR order 1–8 selected by AICc. For the AR(8) models, the Ljung–Box diagnostic used lag 12 with model_df = 8, again leaving four effective degrees of freedom. For an AICc-selected AR(*p*) model, the diagnostic lag was max(8, *p* + 4) and model_df = *p*. Thus, lag 8 was not applied to the AR(8) residual diagnostic. Figure 1 summarizes the analytic framework.

The complete set of supporting analyses and visualizations is provided in Tables S1–S15 and Figures S1–S3.

### 2.6. Reverse, Influence, and Temporal-Stability Analyses

Reverse models exchanged the roles of ILI and SARI while retaining the same lag structure and inferential procedures. Forward and reverse blocks were reported as directional temporal associations, not as causal or exclusive pathways [14].

Seasonal sensitivity analyses omitted Fourier terms or included two annual harmonics. Because 95 weeks do not identify a stable annual pattern, Fourier terms were treated only as parsimonious nuisance adjustment; no seasonal parameter was interpreted. Additional analyses fitted the same lag block to positivity levels, varied the ILI lag window, used AR(8) outcome history, and selected AR order 1–8 by AICc. For the high-change analysis, we removed the model row with the largest absolute SARI change and the row with the largest absolute value among the four lagged ILI changes. We also removed the single row with the largest conventional OLS Cook’s distance and refitted with HAC inference. Supplementary Table S5 gives excluded weeks and final sample sizes.

Temporal variation was described with all 24 overlapping 72-week rolling windows. In each window, we re-estimated the four-lag joint test and cumulative coefficient. These rolling estimates characterize change over calendar time; because adjacent windows share 71 weeks, they are not independent replication samples.

### 2.7. Rolling-Origin Forecast Evaluation

Forecast evaluation examined ΔSARI, because this was the stationary target of the primary model and directly represents whether surveillance activity was increasing or decreasing. The prespecified one-week horizon corresponds to the next weekly surveillance interval. At each origin t − 1, we expanded the training window and predicted week t using SARI and ILI observations available through t − 1 together with calendar terms known for t. This is an idealized operational evaluation: it assumes availability of the preceding week’s ILI and SARI reports before forecast issuance, whereas report-release timestamps were unavailable. Because this target is a first difference rather than a second difference, it is interpreted as a week-to-week increase or decrease, not as acceleration or deceleration.

The initial training set contained the first 52 complete model rows, leaving 38 one-week-ahead forecast origins from 2025-W51 to 2026-W36. Forecasts from the AR(4)-harmonic model plus ILI lags 1–4 were compared with zero-change, expanding-mean, persistence, 52-week seasonal-naive, and AR(4)-harmonic baselines. RMSE and MAE were expressed in percentage points of weekly change. For the augmented-versus-AR comparison, paired circular moving-block bootstrap resampling retained paired errors across 4000 replicates (primary block length 4; seed 20260914), with separate FDR families for MSE and MAE gains. Direct 2- and 4-week-ahead models used the latest four SARI and ILI changes available at each origin and produced 37 and 35 origins, respectively. Each longer-horizon comparison used 3999 paired circular moving-block bootstrap replicates with block length 4. Its 95% percentile interval used quantiles of the uncentered bootstrap distribution, whereas the two-sided *p* value used the absolute centered-null distribution with plus-one correction. Because the interval and test use different bootstrap constructions, they need not be exactly concordant in finite samples. Block lengths 2 and 8 were examined for the one-week analysis [36].

### 2.8. Prewhitened Cross-Correlation

As a secondary descriptive analysis, we fitted AR(*p*) models with *p* = 0–8 to each undifferenced ILI positivity series. We retained models whose ILI residuals passed the Ljung–Box test at 12 lags (*p* > 0.05) and selected the candidate with the lowest AICc. The resulting AR coefficients were applied to the paired undifferenced SARI series [11,37,38]. We defined CCF(k) = corr(filtered ILIt, filtered SARIt + k), so positive k indicates that innovations in ILI preceded innovations in SARI. Model selection used ILI residual whiteness, and filtered SARI diagnostics identified remaining serial dependence. Because eight filtered SARI series remained serially dependent, CCFs and pointwise white-noise limits were treated as descriptive visual summaries; no inferential CCF *p* or q values are reported. This filtered-level analysis and the multivariable first-difference regression estimate different temporal features.

### 2.9. Software

Analyses were conducted in Python 3.12 using NumPy 2.3.5, pandas 2.2.3, SciPy 1.17.0, statsmodels 0.14.4, and direct matrix implementations of ordinary least squares, HAC covariance, Wald tests, and block bootstrap procedures. Figures were produced with Matplotlib 3.10.8. We checked model sample sizes and degrees of freedom, independently recalculated key results, and compared all generated tables with the source workbook.

## 3. Results

### 3.1. Data Completeness and Temporal Patterns

**Figure 2:**
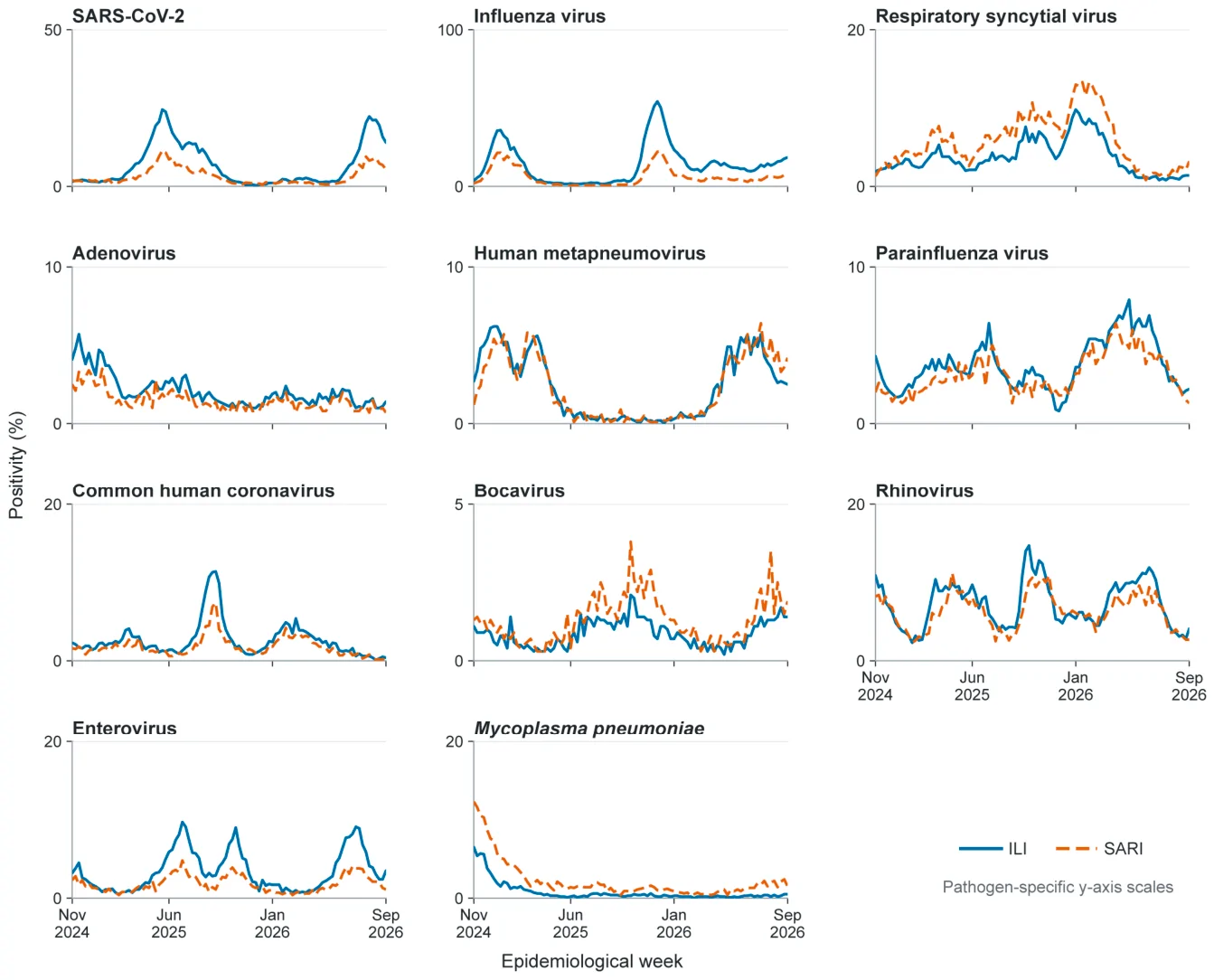
Weekly pathogen positivity in outpatient ILI and hospitalized SARI surveillance. Blue solid lines show ILI positivity and orange dashed lines show SARI positivity from 2024-W46 to 2026-W36. All 95 epidemiological weeks were observed. Each pathogen has its own y-axis scale to preserve lower-amplitude patterns; date labels appear only in the bottom row. Lead–lag associations were evaluated in the distributed-lag models. ILI, influenza-like illness; SARI, severe acute respiratory infection.

The dataset comprised 95 complete weeks, 1045 week-pathogen records, and 2090 positivity values. Each primary dynamic model used 90 observations. Level-series stationarity tests were mixed: the ADF test failed to reject a unit root in 17 of 22 series, while KPSS rejected trend stationarity in 11. After first differencing, ADF rejected a unit root in all 22 series, and KPSS did not reject stationarity in 20; the two *M. pneumoniae* difference series retained conflicting KPSS results (Table S11). Influenza had the highest mean positivity in ILI surveillance (13.82%), whereas rhinovirus had the highest mean positivity in SARI surveillance (6.31%). The pathogen series showed heterogeneous patterns, often with more than one wave (Table 1; Figure 2).

### 3.2. Primary, Cumulative, and Reverse Lag Blocks

The asymptotic joint test of ILI lags 1–4 was FDR-significant for 10 pathogens; bocavirus was not significant (q = 0.925). Among significant blocks, ΔR^2^ ranged from 0.087 to 0.447. Restricted recursive block-bootstrap inference was more selective: influenza, RSV, hMPV, parainfluenza, common human coronavirus, and rhinovirus remained FDR-significant with block lengths 4 and 8, whereas SARS-CoV-2, adenovirus, enterovirus, and *M. pneumoniae* did not (Table 2 and Table S13). The 95-week duration and fewer than two annual cycles therefore limit these estimates to the observed period and do not establish stable pathogen-specific lead times.

The same six pathogens remained FDR-significant across maximum ILI lag windows of 2, 4, 6, and 8 weeks, the level specification, AR(8), and AICc-selected AR specifications (Table S12). Across 24 overlapping rolling 72-week windows, their joint lag blocks were FDR-significant in every window; this describes temporal variation rather than independent replication (Table S15; Figure S3). SARS-CoV-2 was significant in 20 windows, adenovirus and enterovirus in 7 each, *M. pneumoniae* in 4, and bocavirus in none. AICc- and BIC-preferred maximum ILI lags varied by pathogen, supporting retention of the prespecified common four-week window for comparability rather than selection of pathogen-specific lead times.

After FDR correction, cumulative lag effects were significant for SARS-CoV-2, influenza, RSV, parainfluenza, common human coronavirus, rhinovirus, and *M. pneumoniae*. hMPV (q = 0.054) and enterovirus (q = 0.061) did not meet the prespecified threshold; adenovirus and bocavirus were also not significant. Condition numbers ranged from 1.30 to 3.26 and maximum VIFs from 1.06 to 2.59. In the primary AR(4) models, residual serial dependence remained only for enterovirus (Ljung–Box *p* = 0.018). AR(8) removed this signal (*p* = 0.229) but did not improve all pathogens uniformly; residual dependence remained for influenza (*p* = 0.020) and *M. pneumoniae* (*p* = 0.044). HAC inference was therefore retained as covariance correction, not as a substitute for dynamic-model diagnostics (Tables S1 and S12).

SARS-CoV-2 was the only pathogen with FDR-significant asymptotic forward and reverse lag blocks. For nine pathogens, the forward block was significant and the reverse block was not; neither block was significant for bocavirus (Table 2). Reverse-model residual serial dependence remained evident for hMPV (*p* = 0.033), bocavirus (*p* = 0.024), enterovirus (*p* = 0.001), and *M. pneumoniae* (*p* = 0.018) (Table S1). These reverse results are exploratory and do not establish an exclusive direction of effect.

### 3.3. Lag-Specific Findings

**Figure 3:**
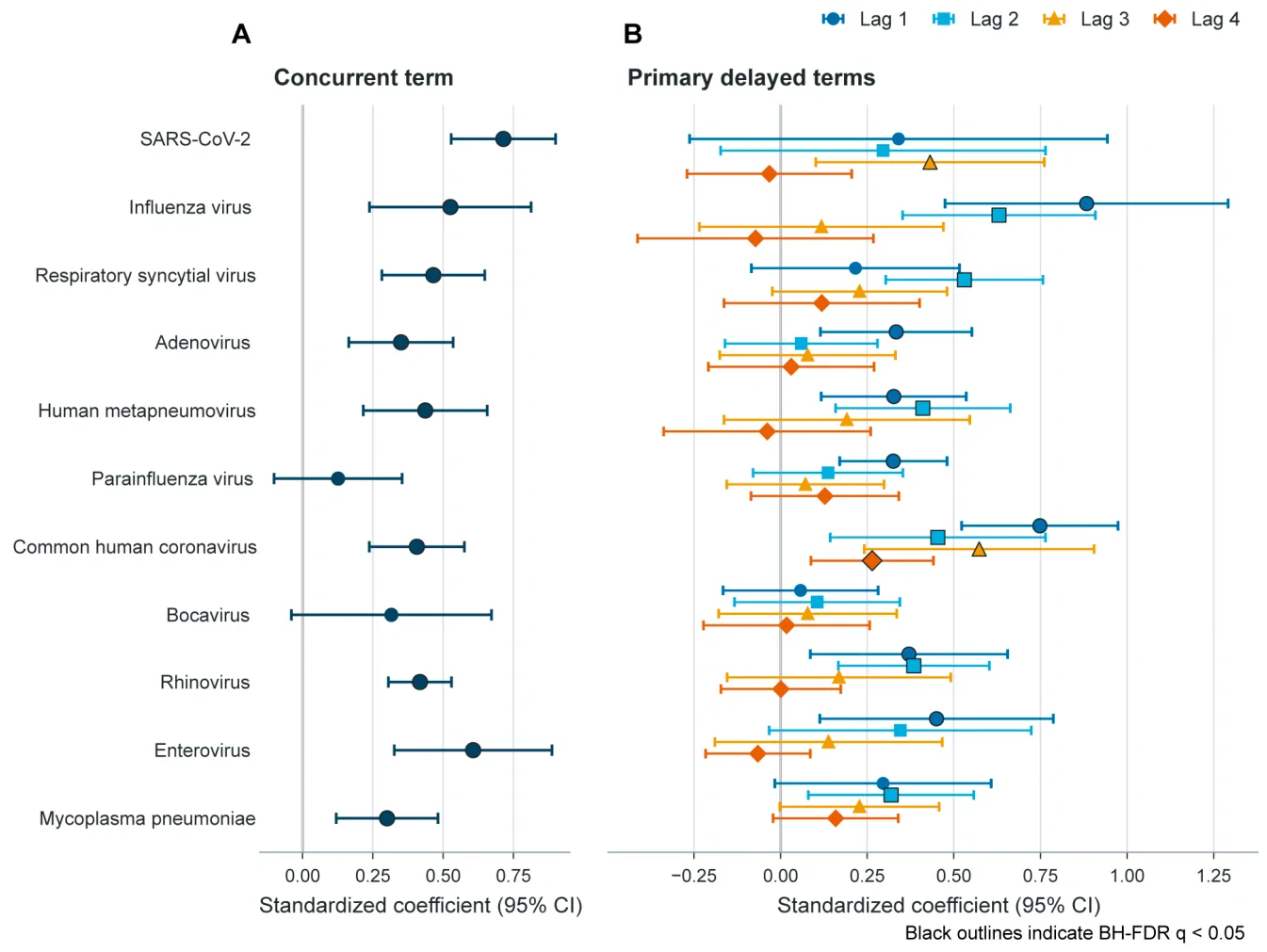
Concurrent and delayed ILI-change coefficients for weekly change in SARI positivity. Points show standardized coefficients, and horizontal lines show 95% confidence intervals based on Newey–West HAC covariance. The vertical gray reference line indicates the null value of zero. Panel (**A**) presents the concurrent lag-0 coefficient from the extended model. Panel (**B**) presents coefficients at lags 1–4 from the primary distributed-lag model; color and marker shape distinguish the four lags. Black outlines indicate BH-FDR *q* < 0.05. *p* values for lag 0 and the delayed terms were adjusted as separate families. The joint test of all four delayed coefficients, rather than any individual lag, was the primary inferential test. ILI, influenza-like illness; SARI, severe acute respiratory infection; HAC, heteroskedasticity- and autocorrelation-consistent; BH-FDR, Benjamini–Hochberg false-discovery rate.

In the exploratory lag-specific analysis, delayed terms remained FDR-significant at lag 3 for SARS-CoV-2; lags 1 and 2 for influenza; lag 2 for RSV; lag 1 for adenovirus; lags 1 and 2 for hMPV; lag 1 for parainfluenza; lags 1–4 for common human coronavirus; lags 1 and 2 for rhinovirus; lag 1 for enterovirus; and lag 2 for *M. pneumoniae*. Concurrent lag-0 terms were FDR-significant for nine pathogens; parainfluenza and bocavirus were the exceptions (Figure 3; Supplementary Table S2).

### 3.4. Rolling-Origin Forecast Performance

**Figure 4:**
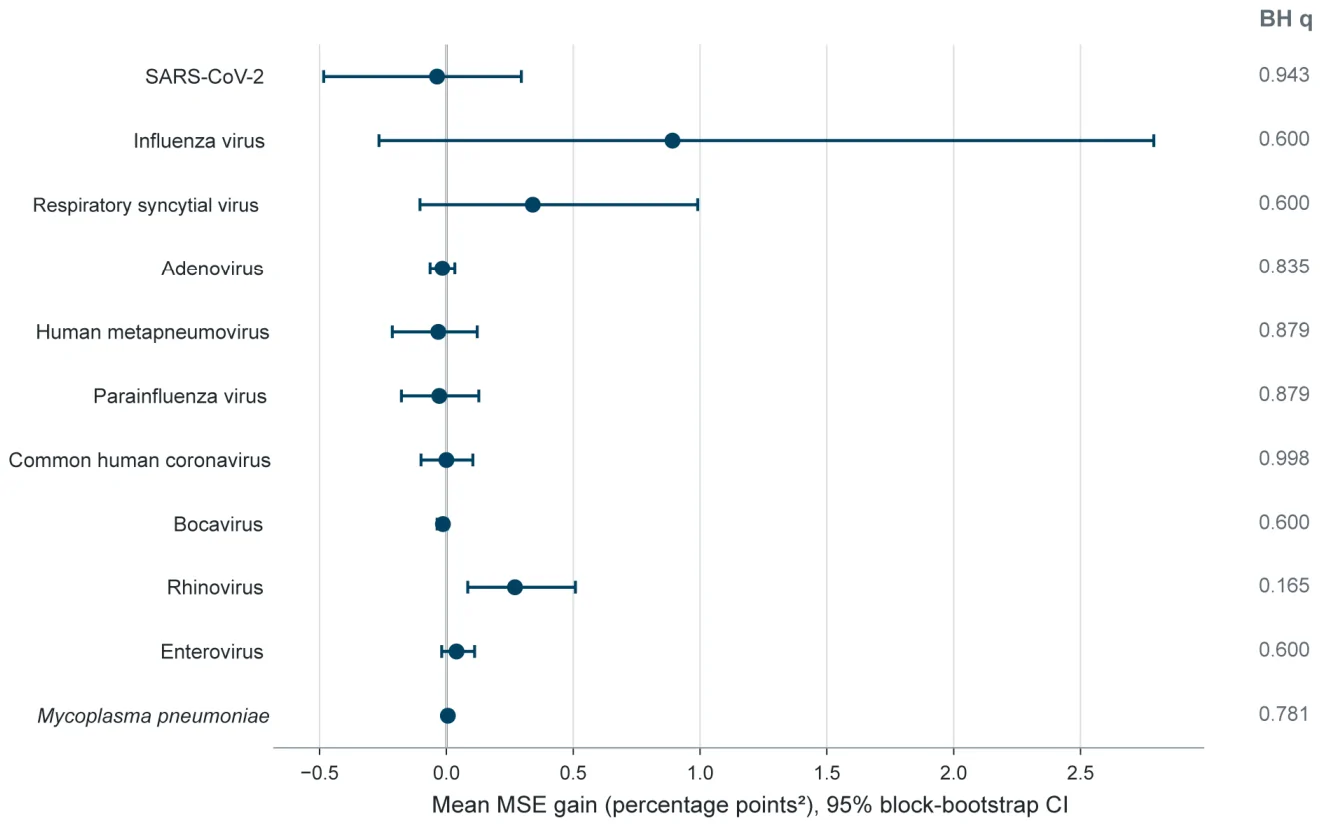
Exploratory incremental value of lagged ILI for one-week-ahead forecasts. Points show the mean difference in mean squared error (MSE) between the autoregressive (AR)-harmonic and augmented AR-harmonic plus ILI models; positive values favor the augmented model. Horizontal lines are paired circular moving-block bootstrap 95% percentile intervals based on block length 4 and 4000 replicates. The vertical gray reference line indicates zero MSE gain. The target was weekly change in SARI positivity, and MSE gain is expressed in squared percentage points. Each pathogen contributed 38 forecast origins. The *q* values displayed at right were adjusted across 11 comparisons using the Benjamini–Hochberg procedure; none was false-discovery-rate-significant. AR, autoregressive; BH, Benjamini–Hochberg; CI, confidence interval; ILI, influenza-like illness; MSE, mean squared error; SARI, severe acute respiratory infection.

At the prespecified one-week horizon, adding lagged ILI lowered RMSE for influenza (23.8%), RSV (15.5%), rhinovirus (13.3%), enterovirus (11.2%), and *M. pneumoniae* (2.2%) and raised RMSE for the other six pathogens. Rhinovirus was the only pathogen with a nominal 95% interval for MSE gain excluding zero, but its adjusted q value was 0.165 across 38 origins; no one-week comparison was FDR-significant. Direct 2- and 4-week analyses likewise found no FDR-significant improvement for any pathogen. At four weeks, parainfluenza forecasts were worse by MAE after FDR correction (gain −0.118 percentage points; q = 0.008), while the MSE comparison did not meet the threshold (q = 0.148) (Tables S6, S7 and S14). Simple baselines matched or outperformed fitted models for several pathogens. Table 3 and Figure 4 present the primary one-week comparison; Figure S2 shows its forecast trajectories.

### 3.5. Cross-Correlation Findings

Contemporaneous correlations between unfiltered levels ranged from r = 0.823 to 0.972 (Table S8). Peak estimates were smaller after ILI-based prewhitening. Eight filtered SARI series retained Ljung–Box evidence of serial dependence, so the CCFs and pointwise white-noise limits are descriptive only, and no inferential *p* or q values are reported. The filtered-level CCFs are not directly comparable with lag coefficients from first-difference multivariable regression (Tables S9 and S10; Figure S1). Among the six pathogens supported by the recursive bootstrap, the maximum positive CCF occurred at lag 0 for influenza, hMPV, and common human coronavirus, and at positive lags for RSV, parainfluenza, and rhinovirus.

## 4. Discussion

### 4.1. Principal Findings and Strength of Evidence

The principal finding was pathogen-specific lagged association in national ILI and SARI positivity. The asymptotic primary test identified 10 lag blocks, but recursive block-bootstrap inference and the broader sensitivity analyses concentrated support on influenza, RSV, hMPV, parainfluenza, common human coronavirus, and rhinovirus. Their associations persisted across prespecified lag windows, level and differenced specifications, alternative outcome-history models, seasonal specifications, influence analyses, and all rolling 72-week windows. These are the strongest in-sample signals in this 95-week dataset; they are not fixed lead-time estimates. SARS-CoV-2, adenovirus, enterovirus, and *M. pneumoniae* had weaker finite-sample support, and bocavirus had no evidence of a lag block. The descriptive CCF findings did not independently corroborate a consistent lead–lag relationship: three of the six bootstrap-supported pathogens had their maximum positive CCF at lag 0. This divergence reinforces that CCFs of filtered levels and multivariable regressions of first differences estimate different temporal features.

The cumulative-effect analysis and low VIFs indicate that severe collinearity among adjacent ILI lags did not account for the joint results. Several significant omnibus blocks had non-significant cumulative sums, showing that the joint test can capture a distributed pattern across lags without a uniform cumulative response. Individual lag coefficients therefore generate temporal hypotheses rather than stable warning intervals.

### 4.2. Forecast Interpretation

Retrospective association did not translate into demonstrated forecast improvement. No pathogen achieved an FDR-significant positive MSE or MAE gain at 1, 2, or 4 weeks. The one-week rhinovirus gain remained nominal, not FDR-significant, across only 38 origins; the longer horizons contained 37 and 35 origins. The significant four-week parainfluenza MAE result favored the AR baseline, not the ILI-augmented model. This separation between strong in-sample association and incremental out-of-sample gain that was not established is the main operational result: lagged ILI positivity should not be treated as a validated standalone forecasting input from the present record. The non-significant comparisons indicate that improvement was not demonstrated in this dataset; they do not prove that the additional predictor has no forecasting value.

### 4.3. Directionality and Biological Interpretation

Forward–reverse contrasts describe temporal ordering within aggregate surveillance systems, not individual disease progression. Influenza, RSV, and hMPV produce recognizable seasonal waves and substantial hospitalization burdens, which can strengthen alignment between outpatient and inpatient positivity. Parainfluenza, common human coronavirus, and rhinovirus may also show coordinated waves across settings, but the apparent timing can depend on age distribution, severity, and care-seeking behavior. Weaker or unstable results for the remaining pathogens may reflect lower weekly positivity, narrower age concentration, episodic circulation, or greater sensitivity to testing composition. These mechanisms are interpretations, not causal findings. Shared weather, school calendars, vaccination, healthcare use, testing, and reporting can generate forward, reverse, or bidirectional blocks; SARS-CoV-2 showed significant asymptotic blocks in both directions [14].

### 4.4. Public Health Implications

ILI and SARI series provide complementary situational awareness, but this 95-week record does not support fixed pathogen-specific lead times or hospital triggers based on ILI positivity alone. Week-to-week change is useful for detecting near-term increases or decreases, yet operational use requires report-availability timestamps, repeated seasons, stable testing volumes and sentinel panels, regional and age strata, and outcomes such as admissions or bed occupancy. Forecast evaluation should retain simple baselines and include release delays [13,39,40,41].

### 4.5. Strengths and Limitations

This study used a common national pathogen panel and separated concurrent from delayed associations. The revised analysis adds formal stationarity diagnostics, level and lag-window comparisons, alternative autoregressive structures, restricted recursive block-bootstrap inference, rolling coefficients, and 2- and 4-week forecast horizons. Reverse-direction models, collinearity diagnostics, seasonal and influence analyses, and descriptive prewhitened CCFs further characterize robustness. Forecasts were evaluated in temporal order against simple baselines, and all numerical results are traceable to the supplied analysis files.

The study has several limitations. Weekly pathogen-specific testing denominators and sentinel-site composition were unavailable; weekly precision may therefore vary, and positivity does not measure incidence or burden. The 95-week series covers fewer than two annual cycles, limiting separation of lagged association from shared seasonality and constraining seasonal, rolling, and forecast estimates. Fourier adjustment and seasonal sensitivity analyses reduce but cannot eliminate this concern. The 24 rolling windows overlap and describe temporal variation rather than independent replication. National aggregation masks heterogeneity by region, age, subtype, vaccination, school calendar, weather, and healthcare use; such shared drivers can generate apparent lead–lag patterns. Forecast analyses included only 38, 37, and 35 origins at 1, 2, and 4 weeks, limiting precision and power to detect modest incremental gains, and assumed that the latest weekly reports were available at each origin. Accordingly, failure to reach the FDR threshold should be interpreted as improvement not established rather than evidence of no forecasting value. Residual dependence was not removed uniformly by a single AR order, and HAC covariance corrects estimated uncertainty rather than the underlying dynamic specification. Finally, eight filtered SARI series remained serially dependent, making CCF white-noise limits approximate visual references. All associations are ecological and temporal rather than causal or individual-level.

## 5. Conclusions

National ILI and SARI positivity showed widespread temporal coupling. Although asymptotic tests identified 10 forward lag blocks, recursive block-bootstrap and specification analyses provided the strongest support for influenza, RSV, hMPV, parainfluenza, common human coronavirus, and rhinovirus. The 95-week series did not establish fixed lead times, and FDR-adjusted evidence that lagged ILI improved forecast accuracy at 1, 2, or 4 weeks was not established in the limited set of forecast origins. Prospective evaluation requires longer stratified series with weekly testing denominators and report-release timestamps.

## Figures and Tables

**Table 1:** Weekly pathogen positivity, 2024-W46 to 2026-W36.

Pathogen	ILI Mean % (Range)	ILI Peak Week(s)	SARI Mean % (Range)	SARI Peak Week(s)
SARS-CoV-2	6.77 (0.3–24.5)	2025-W21	3.20 (0.3–11.5)	2025-W21
Influenza virus	13.82 (1.5–54.2)	2025-W49	6.09 (0.4–22.5)	2025-W50
RSV	3.74 (0.8–9.8)	2026-W02	5.79 (0.8–13.3)	2026-W04, 2026-W06
Adenovirus	2.07 (1.0–5.7)	2024-W48	1.47 (0.7–3.5)	2025-W03
hMPV	2.34 (0.0–6.2)	2024-W52, 2025-W01	2.26 (0.1–6.4)	2026-W28
Parainfluenza virus	3.76 (0.8–7.9)	2026-W18	3.17 (1.3–6.4)	2026-W14
Common human coronavirus	2.64 (0.1–11.4)	2025-W37	1.81 (0.1–7.4)	2025-W37
Bocavirus	0.86 (0.2–2.1)	2025-W41	1.29 (0.2–3.8)	2025-W41
Rhinovirus	7.26 (2.3–14.7)	2025-W40	6.31 (2.3–11.2)	2025-W17
Enterovirus	3.43 (0.5–9.7)	2025-W27	1.82 (0.4–4.8)	2025-W27
*M. pneumoniae*	0.76 (0.0–6.5)	2024-W46	2.18 (0.3–12.3)	2024-W46

All 95 weeks are included. All tied maximum weeks are reported. ILI, influenza-like illness; SARI, severe acute respiratory infection.

**Table 2:** Primary lag-block, cumulative, and reverse-model results.

Pathogen	Forward q	ΔR^2^	Cumulative β (95% CI)	Cumulative q	Reverse q	Bootstrap q
SARS-CoV-2	0.031	0.158	0.68 (0.24, 1.12)	0.007	0.002	0.162
Influenza virus	<0.001	0.352	0.86 (0.40, 1.31)	0.002	0.080	0.003
RSV	<0.001	0.194	1.65 (0.62, 2.68)	0.007	0.199	0.028
Adenovirus	0.039	0.087	0.58 (−0.07, 1.23)	0.088	0.204	0.162
hMPV	<0.001	0.152	0.95 (0.05, 1.86)	0.054	0.152	0.032
Parainfluenza virus	<0.001	0.121	0.74 (0.22, 1.27)	0.010	0.751	0.032
Common human coronavirus	<0.001	0.447	1.66 (0.99, 2.32)	<0.001	0.080	0.008
Bocavirus	0.925	0.004	0.45 (−0.89, 1.79)	0.506	0.204	0.954
Rhinovirus	<0.001	0.233	0.85 (0.26, 1.44)	0.009	0.674	0.023
Enterovirus	0.031	0.105	0.55 (−0.00, 1.10)	0.061	0.080	0.162
*M. pneumoniae*	0.039	0.105	1.54 (0.53, 2.55)	0.007	0.080	0.345

Forward q values are BH-FDR-adjusted asymptotic HAC-Wald results; bootstrap q values use 3999 restricted recursive circular moving-block replicates with block length 4. Cumulative β is the sum of coefficients at lags 1–4. Its 95% CI is unadjusted, whereas q values are FDR-adjusted. Supplementary Table S2 reports exploratory individual-lag estimates.

**Table 3:** One-week-ahead forecast comparison.

Pathogen	AR RMSE	+ILI RMSE	RMSE Change	Mean MSE Gain (95% CI)	*p*	q
SARS-CoV-2	0.913	0.933	−2.2%	−0.037 (−0.484, 0.296)	0.857	0.943
Influenza virus	1.458	1.110	+23.8%	0.891 (−0.266, 2.789)	0.273	0.600
RSV	1.090	0.921	+15.5%	0.340 (−0.104, 0.991)	0.227	0.600
Adenovirus	0.382	0.402	−5.3%	−0.016 (−0.064, 0.033)	0.531	0.835
hMPV	0.774	0.795	−2.6%	−0.032 (−0.213, 0.122)	0.704	0.879
Parainfluenza virus	0.658	0.679	−3.2%	−0.028 (−0.177, 0.128)	0.720	0.879
Common human coronavirus	0.485	0.485	−0.0%	−0.000 (−0.100, 0.104)	0.998	0.998
Bocavirus	0.440	0.456	−3.5%	−0.014 (−0.036, 0.004)	0.194	0.600
Rhinovirus	1.045	0.906	+13.3%	0.270 (0.084, 0.509)	0.015	0.165
Enterovirus	0.434	0.385	+11.2%	0.040 (−0.019, 0.112)	0.232	0.600
*M. pneumoniae*	0.368	0.360	+2.2%	0.006 (−0.009, 0.020)	0.426	0.781

The prediction target was weekly change in SARI positivity, expressed in percentage points; MSE gains are in squared percentage points. Positive gains favor the AR-harmonic model plus ILI. Each comparison used the same 38 forecast origins. *p* values were obtained from the centered circular block bootstrap with block length 4, and q values were adjusted across 11 pathogens with the Benjamini–Hochberg procedure.

## Data Availability

The weekly surveillance reports used in this study are publicly available through the China CDC National Acute Respiratory Infectious Disease Sentinel Surveillance portal (https://www.chinacdc.cn/jksj/jksj04_14275/, accessed on 12 September 2026) [29]. The processed analytic dataset, source register, quality-control log, analysis code, and generated revision outputs are included in Supplementary File S1 and will be publicly accessible with the published article.

## Supplementary Materials

The following supporting information can be downloaded at: https://www.mdpi.com/article/10.3390/pathogens15101081/s1. Table S1: Complete forward and reverse lag-block results; Table S2: Concurrent and lag-specific coefficients; Table S3: Cumulative lag effects and collinearity diagnostics; Table S4: FDR-adjusted sensitivity-analysis *q* values; Table S5: Influence-analysis algorithms and removed weeks; Table S6: Forecast accuracy across comparator models; Table S7: Paired bootstrap forecast-gain estimates and block-length sensitivity; Table S8: Descriptive raw contemporaneous correlations of positivity levels; Table S9: Peak prewhitened cross-correlations; Table S10: Prewhitening diagnostics; Table S11: Stationarity diagnostics for positivity levels and first differences; Table S12a: Sensitivity to the maximum ILI lag window; Table S12b: Sensitivity to level and alternative outcome-history specifications; Table S13: Restricted recursive moving-block bootstrap sensitivity for the primary forward lag block; Table S14a: Direct 2- and 4-week forecast comparisons based on mean squared error; Table S14b: Direct 2- and 4-week forecast comparisons based on mean absolute error; Table S15: Summary of cumulative coefficients across all rolling 72-week windows; Figure S1: Prewhitened cross-correlations between ILI and SARI pathogen positivity; Figure S2: Observed and rolling-origin predicted weekly changes in SARI positivity; Figure S3: Rolling 72-week cumulative lag coefficients; Supplementary File S1: Reproducibility package containing the processed dataset, source register, quality-control materials, analysis code, and generated outputs.

## Abbreviations

AICc, corrected Akaike information criterion; AR, autoregressive; BH-FDR, Benjamini–Hochberg false-discovery rate; CCF, cross-correlation function; CI, confidence interval; HAC, heteroskedasticity- and autocorrelation-consistent; hMPV, human metapneumovirus; ILI, influenza-like illness; MAE, mean absolute error; MSE, mean squared error; OLS, ordinary least squares; RMSE, root mean squared error; RSV, respiratory syncytial virus; SARI, severe acute respiratory infection; SARS-CoV-2, severe acute respiratory syndrome coronavirus 2; STROBE, Strengthening the Reporting of Observational Studies in Epidemiology; VIF, variance-inflation factor.
