# Supplementary material for: Pathogen-Specific Temporal Coupling Between Outpatient Influenza-like Illness and Hospitalized Severe Acute Respiratory Infection Positivity in China: A National Sentinel Surveillance Time-Series Study
Source: Pathogens. 2026 Oct 14;15(10):1081. doi: 10.3390/pathogens15101081 (PMC13708613; doi:10.3390/pathogens15101081)
Supplement: Supplementary file 1 [file pathogens-15-01081-s001.zip › Supplementary Tables and Figures.pdf]

Supplementary Materials

Pathogen-Specific Temporal Coupling between Outpatient Influenza-Like Illness and Hospitalized Severe Acute Respiratory Infection Positivity in China

Qiong Chen, Shanshan Tong, Jun Gao and Sufang Xu

This file contains Supplementary Tables S1-S15 and Supplementary Figures S1-S3.

Table S1. Complete forward and reverse lag-block results

Pathogen	N	Forward F	df	Raw P	q	ΔR²	Forward LB P	Reverse F	Raw P	q	Reverse LB P
SARS-CoV-2	90	3.015	4,79	0.023	0.031	0.158	0.431	6.437	<0.001	0.002	0.298
Influenza virus	90	22.859	4,79	<0.001	<0.001	0.352	0.368	2.969	0.024	0.080	0.508
RSV	90	5.933	4,79	<0.001	<0.001	0.194	0.489	1.856	0.126	0.199	0.195
Adenovirus	90	2.778	4,79	0.032	0.039	0.087	0.105	1.667	0.166	0.204	0.356
hMPV	90	5.958	4,79	<0.001	<0.001	0.152	0.682	2.146	0.083	0.152	0.033
Parainfluenza virus	90	5.599	4,79	<0.001	<0.001	0.121	0.124	0.479	0.751	0.751	0.055
Common human corona-virus	90	12.155	4,79	<0.001	<0.001	0.447	0.133	2.700	0.036	0.080	0.443
Bocavirus	90	0.222	4,79	0.925	0.925	0.004	0.431	1.664	0.167	0.204	0.024
Rhinovirus	90	8.654	4,79	<0.001	<0.001	0.233	0.193	0.673	0.612	0.674	0.599
Enterovirus	90	3.122	4,79	0.019	0.031	0.105	0.018	2.812	0.031	0.080	0.001
M. pneumoniae	90	2.726	4,79	0.035	0.039	0.105	0.293	3.218	0.017	0.080	0.018

The Wald F test jointly evaluates four cross-series lags. Both directions have 79 residual degrees of freedom. Ljung-Box tests at lag 8 used 4 diagnostic degrees of freedom after accounting for four autoregressive outcome-lag coefficients (8 - 4 = 4). Forward and reverse q values were adjusted in separate families of 11 tests.

Table S2. Concurrent and lag-specific coefficients

Pathogen	Lag	β	HAC SE	CI low	CI high	Std β	Std CI low	Std CI high	Raw P	q
SARS-CoV-2	0	0.4481	0.0585	0.3315	0.5646	0.7143	0.5286	0.9001	<0.001	<0.001
SARS-CoV-2	1	0.2156	0.1918	-0.1662	0.5975	0.3405	-0.2625	0.9436	0.264	0.447
SARS-CoV-2	2	0.1950	0.1553	-0.1142	0.5042	0.2956	-0.1731	0.7644	0.213	0.391
SARS-CoV-2	3	0.2877	0.1105	0.0678	0.5075	0.4315	0.1017	0.7614	0.011	0.032
SARS-CoV-2	4	-0.0216	0.0796	-0.1800	0.1368	-0.0324	-0.2700	0.2052	0.787	0.837
Influenza virus	0	0.2938	0.0807	0.1331	0.4544	0.5256	0.2381	0.8132	<0.001	<0.001

Pathogen	Lag	β	HAC SE	CI low	CI high	Std β	Std CI low	Std CI high	Raw P	q
Influenza virus	1	0.4857	0.1129	0.2610	0.7104	0.8833	0.4747	1.2920	<0.001	<0.001
Influenza virus	2	0.3446	0.0764	0.1925	0.4967	0.6305	0.3522	0.9087	<0.001	<0.001
Influenza virus	3	0.0645	0.0966	-0.1278	0.2567	0.1180	-0.2340	0.4700	0.507	0.667
Influenza virus	4	-0.0395	0.0933	-0.2252	0.1463	-0.0723	-0.4126	0.2680	0.674	0.760
RSV	0	0.7041	0.1393	0.4267	0.9815	0.4651	0.2818	0.6483	<0.001	<0.001
RSV	1	0.3260	0.2275	-0.1268	0.7788	0.2162	-0.0841	0.5165	0.156	0.312
RSV	2	0.8001	0.1720	0.4577	1.1426	0.5306	0.3035	0.7577	<0.001	<0.001
RSV	3	0.3445	0.1913	-0.0363	0.7253	0.2281	-0.0240	0.4802	0.076	0.166
RSV	4	0.1797	0.2139	-0.2461	0.6055	0.1190	-0.1630	0.4011	0.403	0.555
Adenovirus	0	0.4174	0.1112	0.1960	0.6388	0.3500	0.1644	0.5356	<0.001	<0.001
Adenovirus	1	0.3917	0.1290	0.1350	0.6485	0.3337	0.1150	0.5525	0.003	0.014
Adenovirus	2	0.0678	0.1254	-0.1818	0.3173	0.0598	-0.1603	0.2798	0.590	0.702
Adenovirus	3	0.0862	0.1407	-0.1938	0.3662	0.0781	-0.1755	0.3316	0.542	0.667
Adenovirus	4	0.0337	0.1319	-0.2288	0.2962	0.0307	-0.2085	0.2700	0.799	0.837
hMPV	0	0.4712	0.1195	0.2334	0.7090	0.4363	0.2161	0.6565	<0.001	<0.001
hMPV	1	0.3518	0.1132	0.1264	0.5771	0.3267	0.1174	0.5360	0.003	0.013
hMPV	2	0.4419	0.1362	0.1707	0.7130	0.4107	0.1586	0.6627	0.002	0.010
hMPV	3	0.1996	0.1857	-0.1701	0.5692	0.1914	-0.1631	0.5460	0.286	0.466
hMPV	4	-0.0399	0.1556	-0.3497	0.2698	-0.0385	-0.3371	0.2602	0.798	0.837
Parainfluenza virus	0	0.1426	0.1293	-0.1148	0.4000	0.1262	-0.1016	0.3540	0.274	0.274
Parainfluenza virus	1	0.3671	0.0878	0.1924	0.5418	0.3253	0.1705	0.4801	<0.001	<0.001
Parainfluenza virus	2	0.1541	0.1223	-0.0893	0.3976	0.1370	-0.0794	0.3534	0.211	0.391
Parainfluenza virus	3	0.0802	0.1276	-0.1738	0.3342	0.0716	-0.1552	0.2985	0.531	0.667
Parainfluenza virus	4	0.1423	0.1193	-0.0951	0.3798	0.1279	-0.0854	0.3412	0.236	0.416
Common human coronavirus	0	0.3304	0.0690	0.1931	0.4677	0.4065	0.2375	0.5754	<0.001	<0.001
Common human coronavirus	1	0.6079	0.0921	0.4246	0.7912	0.7485	0.5228	0.9742	<0.001	<0.001
Common human coronavirus	2	0.3688	0.1268	0.1163	0.6212	0.4539	0.1431	0.7647	0.005	0.017

Pathogen	Lag	β	HAC SE	CI low	CI high	Std β	Std CI low	Std CI high	Raw P	q
Common human coronavirus	3	0.4653	0.1353	0.1961	0.7346	0.5732	0.2416	0.9049	<0.001	0.006
Common human coronavirus	4	0.2147	0.0722	0.0711	0.3583	0.2644	0.0875	0.4412	0.004	0.016
Bocavirus	0	0.5466	0.3094	-0.0694	1.1626	0.3158	-0.0401	0.6717	0.081	0.089
Bocavirus	1	0.0998	0.1946	-0.2875	0.4872	0.0577	-0.1662	0.2816	0.609	0.706
Bocavirus	2	0.1838	0.2088	-0.2317	0.5993	0.1056	-0.1332	0.3444	0.381	0.555
Bocavirus	3	0.1369	0.2258	-0.3125	0.5863	0.0782	-0.1787	0.3352	0.546	0.667
Bocavirus	4	0.0303	0.2104	-0.3884	0.4490	0.0174	-0.2225	0.2572	0.886	0.906
Rhinovirus	0	0.3855	0.0520	0.2819	0.4891	0.4175	0.3053	0.5297	<0.001	<0.001
Rhinovirus	1	0.3442	0.1330	0.0795	0.6088	0.3706	0.0857	0.6556	0.011	0.032
Rhinovirus	2	0.3513	0.1000	0.1522	0.5504	0.3846	0.1667	0.6026	<0.001	0.005
Rhinovirus	3	0.1539	0.1481	-0.1408	0.4486	0.1685	-0.1542	0.4913	0.302	0.474
Rhinovirus	4	0.0008	0.0788	-0.1560	0.1577	0.0009	-0.1721	0.1739	0.992	0.992
Enterovirus	0	0.3862	0.0898	0.2075	0.5649	0.6066	0.3259	0.8874	<0.001	<0.001
Enterovirus	1	0.2891	0.1087	0.0727	0.5056	0.4501	0.1132	0.7870	0.009	0.030
Enterovirus	2	0.2159	0.1187	-0.0204	0.4523	0.3455	-0.0326	0.7236	0.073	0.166
Enterovirus	3	0.0864	0.1029	-0.1184	0.2911	0.1384	-0.1896	0.4664	0.404	0.555
Enterovirus	4	-0.0409	0.0475	-0.1355	0.0536	-0.0653	-0.2163	0.0857	0.392	0.555
M. pneumoniae	0	0.6308	0.1910	0.2506	1.0110	0.3003	0.1193	0.4813	0.001	0.002
M. pneumoniae	1	0.4596	0.2436	-0.0253	0.9445	0.2959	-0.0163	0.6080	0.063	0.154
M. pneumoniae	2	0.4980	0.1872	0.1255	0.8705	0.3190	0.0804	0.5577	0.009	0.030
M. pneumoniae	3	0.3531	0.1789	-0.0030	0.7092	0.2279	-0.0020	0.4578	0.052	0.134
M. pneumoniae	4	0.2294	0.1308	-0.0309	0.4897	0.1592	-0.0215	0.3398	0.083	0.175

Lag 0 was estimated in the extended synchronous model; lags 1-4 were estimated in the primary model. Standardized confidence limits correspond to standardized coefficients in Figure 3. Individual delayed lags were exploratory. P values for lag 0 and delayed coefficients were adjusted as separate families.

Table S3. Cumulative lag effects and collinearity diagnostics

Pathogen	Σβ	CI low	CI high	Raw P	q	Condition no.	Max VIF	VIF lag 1/2/3/4
SARS-CoV-2	0.677	0.235	1.118	0.003	0.007	1.30	1.06	1.05/1.04/1.03/1.06

Pathogen	Σβ	CI low	CI high	Raw P	q	Condition no.	Max VIF	VIF lag 1/2/3/4
Influenza virus	0.855	0.401	1.309	<0.001	0.002	1.45	1.14	1.11/1.14/1.02/1.02
RSV	1.650	0.622	2.679	0.002	0.007	1.58	1.16	1.06/1.09/1.16/1.12
Adenovirus	0.579	-0.071	1.230	0.080	0.088	1.84	1.32	1.17/1.30/1.32/1.19
hMPV	0.953	0.050	1.857	0.039	0.054	1.79	1.29	1.15/1.29/1.29/1.14
Parainfluenza virus	0.744	0.218	1.269	0.006	0.010	1.55	1.14	1.11/1.13/1.14/1.11
Common human corona-virus	1.657	0.990	2.323	<0.001	<0.001	1.36	1.08	1.01/1.06/1.08/1.05
Bocavirus	0.451	-0.893	1.794	0.506	0.506	3.26	2.59	1.65/2.59/2.59/1.65
Rhinovirus	0.850	0.264	1.436	0.005	0.009	1.53	1.16	1.16/1.12/1.09/1.09
Enterovirus	0.551	-0.001	1.102	0.050	0.061	1.68	1.28	1.07/1.28/1.20/1.05
M. pneumoniae	1.540	0.528	2.552	0.003	0.007	1.61	1.16	1.09/1.13/1.16/1.15

The cumulative effect is the sum of the four delayed coefficients. Its 95% CI is unadjusted, whereas q values are FDR-adjusted. It represents a one-percentage-point increase in ILI positivity at each of lags 1-4, not the effect of a single one-week increase. Before calculating condition numbers and VIFs, each ILI lag was residualized on the SARI lags and Fourier terms.

Table S4. FDR-adjusted sensitivity-analysis q values

Pathogen	No Fourier	Two harmon-ics	HAC(8)	First 72	Last 72	Remove high changes	Remove max Cook
Adenovirus	0.036	0.069	0.027	0.038	0.226	0.282	0.059
Bocavirus	0.614	0.923	0.876	0.706	0.858	0.989	0.400
Common human coronavirus	<0.001	<0.001	<0.001	<0.001	<0.001	<0.001	<0.001
Enterovirus	0.014	0.007	0.027	0.332	0.016	0.030	0.059
Influenza virus	<0.001	<0.001	<0.001	<0.001	<0.001	<0.001	<0.001
M. pneumoniae	0.038	0.007	0.045	0.038	0.052	0.161	0.105
Parainfluenza virus	<0.001	0.001	<0.001	0.002	<0.001	0.013	0.001
RSV	<0.001	<0.001	<0.001	<0.001	0.015	<0.001	<0.001
Rhinovirus	<0.001	<0.001	<0.001	<0.001	<0.001	0.002	<0.001
SARS-CoV-2	0.014	0.015	0.007	<0.001	0.052	<0.001	0.002
hMPV	<0.001	<0.001	<0.001	<0.001	<0.001	0.003	<0.001

Each column was adjusted across 11 pathogens using the Benjamini-Hochberg procedure. The first and last 72-week windows overlap and are sensitivity analyses, not independent validation samples; all 24 rolling 72-week estimates are shown in Table S15 and Figure S3.

Table S5. Influence-analysis algorithms and removed weeks

Pathogen	Analysis	Final N	Removed model week(s)	Cook D	F	Raw P	q
SARS-CoV-2	Remove largest ILI/SARI changes	89	2026-W30		6.056	<0.001	<0.001
SARS-CoV-2	Remove highest Cook's distance	89	2025-W23	0.618	5.063	0.001	0.002
Influenza virus	Remove largest ILI/SARI changes	88	2025-W45; 2025-W46		16.869	<0.001	<0.001
Influenza virus	Remove highest Cook's distance	89	2025-W04	0.418	52.924	<0.001	<0.001
RSV	Remove largest ILI/SARI changes	88	2025-W37; 2025-W38		6.594	<0.001	<0.001
RSV	Remove highest Cook's distance	89	2025-W40	0.158	10.382	<0.001	<0.001
Adenovirus	Remove largest ILI/SARI changes	88	2025-W03; 2025-W19		1.358	0.257	0.282
Adenovirus	Remove highest Cook's distance	89	2025-W05	0.250	2.544	0.046	0.059
hMPV	Remove largest ILI/SARI changes	88	2026-W21; 2026-W29		4.880	0.001	0.003
hMPV	Remove highest Cook's distance	89	2026-W31	0.264	6.831	<0.001	<0.001
Parainfluenza virus	Remove largest ILI/SARI changes	88	2025-W28; 2026-W20		3.682	0.009	0.013
Parainfluenza virus	Remove highest Cook's distance	89	2025-W28	0.153	5.331	<0.001	0.001
Common human corona-virus	Remove largest ILI/SARI changes	88	2025-W38; 2025-W40		9.291	<0.001	<0.001
Common human corona-virus	Remove highest Cook's distance	89	2025-W38	1.106	13.879	<0.001	<0.001
Bocavirus	Remove largest ILI/SARI changes	88	2025-W42; 2026-W32		0.078	0.989	0.989
Bocavirus	Remove highest Cook's distance	89	2025-W41	0.226	1.025	0.400	0.400
Rhinovirus	Remove largest ILI/SARI changes	88	2025-W39; 2025-W47		5.170	<0.001	0.002
Rhinovirus	Remove highest Cook's distance	89	2025-W17	0.320	18.923	<0.001	<0.001
Enterovirus	Remove largest ILI/SARI changes	88	2025-W27; 2025-W45		3.040	0.022	0.030
Enterovirus	Remove highest Cook's distance	89	2025-W29	0.258	2.513	0.048	0.059

Pathogen	Analysis	Final N	Removed model week(s)	Cook D	F	Raw P	q
M. pneumoniae	Remove largest ILI/SARI changes	88	2024-W51; 2025-W01		1.829	0.132	0.161
M. pneumoniae	Remove highest Cook's distance	89	2024-W51	0.544	2.048	0.096	0.105

The high-change analysis removed the row with the largest absolute outcome change and the row with the largest absolute value among the four lagged ILI changes. If both criteria identified the same row, it was removed once. For the Cook's-distance analysis, we removed the row with the largest conventional OLS Cook's D and then refitted the model using HAC inference.

Table S6. Forecast accuracy across comparator models

Pathogen	Zero RMSE	Mean RMSE	Persist. RMSE	Seasonal RMSE	AR RMSE	AR+ILI RMSE	AR MAE	AR+ILI MAE
Adenovirus	0.387	0.391	0.653	0.623	0.382	0.402	0.308	0.298
Bocavirus	0.539	0.543	0.922	0.767	0.440	0.456	0.315	0.328
Common human corona-virus	0.487	0.491	0.747	0.802	0.485	0.485	0.376	0.413
Enterovirus	0.457	0.460	0.678	0.686	0.434	0.385	0.344	0.305
Influenza virus	1.480	1.526	1.464	2.817	1.458	1.110	1.091	0.924
M. pneumoniae	0.371	0.394	0.622	0.620	0.368	0.360	0.281	0.272
Parainfluenza virus	0.670	0.675	1.069	0.895	0.658	0.679	0.555	0.579
RSV	1.029	1.040	1.320	1.330	1.090	0.921	0.774	0.722
Rhinovirus	1.053	1.057	1.744	1.466	1.045	0.906	0.850	0.732
SARS-CoV-2	0.961	0.966	1.469	1.415	0.913	0.933	0.629	0.622
hMPV	0.691	0.700	1.090	1.101	0.774	0.795	0.579	0.588

All metrics describe the same 38 one-week-ahead forecasts of weekly change in SARI positivity and are expressed in percentage points. The seasonal-naive forecast uses the change observed 52 weeks earlier.

Table S7. Paired bootstrap forecast-gain estimates and block-length sensitivity

Pathogen	Block L	MSE gain	MSE 95% CI	Raw P	MAE gain	MAE 95% CI	Raw P	MSE q (L=4)	MAE q (L=4)
SARS-CoV-2	2	-0.0369	-0.5936 to 0.3537	0.896	0.0072	-0.1560 to 0.1204	0.924	-	-
SARS-CoV-2	4	-0.0369	-0.4836 to 0.2955	0.857	0.0072	-0.1318 to 0.1128	0.915	0.943	0.915
SARS-CoV-2	8	-0.0369	-0.3558 to 0.2375	0.807	0.0072	-0.0864 to 0.0911	0.874	-	-
Influenza virus	2	0.8914	-0.2922 to 2.7419	0.259	0.1672	-0.1090 to 0.5165	0.292	-	-
Influenza virus	4	0.8914	-0.2658 to 2.7885	0.273	0.1672	-0.1097 to 0.5531	0.324	0.600	0.594

Pathogen	Block L	MSE gain	MSE 95% CI	Raw P	MAE gain	MAE 95% CI	Raw P	MSE q (L=4)	MAE q (L=4)
Influenza virus	8	0.8914	-0.1652 to 2.6583	0.252	0.1672	-0.0744 to 0.4788	0.252	-	-
RSV	2	0.3404	-0.1040 to 0.9313	0.187	0.0524	-0.1027 to 0.2270	0.530	-	-
RSV	4	0.3404	-0.1039 to 0.9908	0.227	0.0524	-0.1023 to 0.2542	0.577	0.600	0.876
RSV	8	0.3404	-0.0614 to 0.9812	0.215	0.0524	-0.1103 to 0.2494	0.595	-	-
Adenovirus	2	-0.0159	-0.0615 to 0.0261	0.469	0.0094	-0.0390 to 0.0561	0.692	-	-
Adenovirus	4	-0.0159	-0.0641 to 0.0329	0.531	0.0094	-0.0411 to 0.0632	0.716	0.835	0.876
Adenovirus	8	-0.0159	-0.0638 to 0.0378	0.545	0.0094	-0.0376 to 0.0648	0.733	-	-
hMPV	2	-0.0320	-0.2217 to 0.1368	0.724	-0.0091	-0.1120 to 0.0853	0.848	-	-
hMPV	4	-0.0320	-0.2130 to 0.1216	0.704	-0.0091	-0.1040 to 0.0739	0.839	0.879	0.915
hMPV	8	-0.0320	-0.2045 to 0.1022	0.716	-0.0091	-0.1008 to 0.0658	0.843	-	-
Parainfluenza virus	2	-0.0281	-0.1724 to 0.1286	0.719	-0.0235	-0.1301 to 0.0960	0.686	-	-
Parainfluenza virus	4	-0.0281	-0.1773 to 0.1279	0.720	-0.0235	-0.1311 to 0.1013	0.687	0.879	0.876
Parainfluenza virus	8	-0.0281	-0.2040 to 0.1217	0.743	-0.0235	-0.1320 to 0.1044	0.703	-	-
Common human coro-navirus	2	-0.0001	-0.1067 to 0.1256	1.000	-0.0366	-0.1188 to 0.0426	0.380	-	-
Common human coro-navirus	4	-0.0001	-0.0999 to 0.1044	0.998	-0.0366	-0.1083 to 0.0284	0.291	0.998	0.594
Common human coro-navirus	8	-0.0001	-0.0744 to 0.0861	0.998	-0.0366	-0.0901 to 0.0183	0.183	-	-
Bocavirus	2	-0.0138	-0.0317 to 0.0012	0.103	-0.0131	-0.0367 to 0.0061	0.236	-	-
Bocavirus	4	-0.0138	-0.0363 to 0.0038	0.194	-0.0131	-0.0426 to 0.0096	0.319	0.600	0.594
Bocavirus	8	-0.0138	-0.0393 to 0.0040	0.260	-0.0131	-0.0481 to 0.0110	0.419	-	-
Rhinovirus	2	0.2702	0.0838 to 0.4869	0.010	0.1179	0.0294 to 0.2035	0.007	-	-
Rhinovirus	4	0.2702	0.0840 to 0.5088	0.015	0.1179	0.0330 to 0.1987	0.006	0.165	0.066
Rhinovirus	8	0.2702	0.0849 to 0.4985	0.010	0.1179	0.0326 to 0.2003	0.005	-	-
Enterovirus	2	0.0398	-0.0137 to 0.1046	0.181	0.0395	-0.0101 to 0.0970	0.138	-	-
Enterovirus	4	0.0398	-0.0189 to 0.1115	0.232	0.0395	-0.0130 to 0.1002	0.174	0.600	0.594
Enterovirus	8	0.0398	-0.0235 to 0.1089	0.266	0.0395	-0.0075 to 0.0946	0.136	-	-
M. pneumoniae	2	0.0058	-0.0105 to 0.0227	0.489	0.0094	-0.0095 to 0.0287	0.330	-	-
M. pneumoniae	4	0.0058	-0.0091 to 0.0200	0.426	0.0094	-0.0095 to 0.0270	0.297	0.781	0.594

Pathogen	Block L	MSE gain	MSE 95% CI	Raw P	MAE gain	MAE 95% CI	Raw P	MSE q (L=4)	MAE q (L=4)
M. pneumoniae	8	0.0058	-0.0065 to 0.0177	0.351	0.0094	-0.0075 to 0.0250	0.263	-	-

Positive gains favor the AR-harmonic model plus ILI. Percentile intervals were obtained from 4000 paired circular moving-block bootstrap replicates. Two-sided raw P values were calculated under a centered bootstrap null distribution. Block length 4 was prespecified as the primary bootstrap analysis; its MSE-gain and MAE-gain P values were adjusted across pathogens as separate families.

Table S8. Descriptive raw contemporaneous correlations of positivity levels

Pathogen	N	Pearson r	95% CI low	95% CI high
SARS-CoV-2	95	0.972	0.958	0.981
Influenza virus	95	0.933	0.901	0.955
RSV	95	0.951	0.927	0.967
Adenovirus	95	0.850	0.783	0.898
hMPV	95	0.933	0.901	0.955
Parainfluenza virus	95	0.858	0.794	0.903
Common human coronavirus	95	0.924	0.888	0.949
Bocavirus	95	0.823	0.746	0.879
Rhinovirus	95	0.858	0.794	0.904
Enterovirus	95	0.932	0.899	0.954
M. pneumoniae	95	0.972	0.957	0.981

Fisher-z intervals describe raw contemporaneous correlations and do not account for autocorrelation or seasonality.

Table S9. Peak prewhitened cross-correlations

Pathogen	Lag of max CCF	r	Max |r| lag	Signed r	Best ILI-leading lag 0-8	r
SARS-CoV-2	-1	0.246	-1	0.246	0	0.182
Influenza virus	0	0.351	0	0.351	0	0.351
Respiratory syncytial virus	2	0.305	2	0.305	2	0.305
Adenovirus	0	0.293	0	0.293	0	0.293
Human metapneumovirus	0	0.252	8	-0.330	0	0.252
Parainfluenza virus	1	0.320	-6	-0.329	1	0.320

Pathogen	Lag of max CCF	r	Max |r| lag	Signed r	Best ILI-leading lag 0-8	r
Common human corona-virus	0	0.346	0	0.346	0	0.346
Bocavirus	0	0.327	0	0.327	0	0.327
Rhinovirus	2	0.291	2	0.291	2	0.291
Enterovirus	-1	0.277	-6	-0.284	0	0.255
Mycoplasma pneumoniae	-8	0.328	-8	0.328	0	0.207

The maximum CCF is the largest signed r between -8 and +8 weeks. The maximum absolute CCF is the largest |r| over the same range, reported with its sign. Positive lags indicate that ILI preceded SARI. Because eight filtered SARI series retained residual serial dependence, these CCF summaries are descriptive and no inferential P or q values are reported.

Table S10. Prewhitening diagnostics

Pathogen	d	AR p	Effective N	AICc	ILI residual LB P	SARI filtered LB P
SARS-CoV-2	0	2	93	27.55	0.413	<0.001
Influenza virus	0	6	89	130.12	0.710	<0.001
Respiratory syncytial virus	0	3	92	-69.30	0.453	0.687
Adenovirus	0	3	92	-174.63	0.639	<0.001
Human metapneumovirus	0	3	92	-95.47	0.211	0.920
Parainfluenza virus	0	4	91	-97.09	0.363	0.197
Common human coronavirus	0	3	92	-57.35	0.526	<0.001
Bocavirus	0	2	93	-246.22	0.341	0.044
Rhinovirus	0	6	89	18.73	0.114	<0.001
Enterovirus	0	4	91	-47.25	0.539	<0.001
Mycoplasma pneumoniae	0	4	91	-324.69	0.241	0.001

Undifferenced levels were filtered without differencing (d=0). AR order was selected from whiteness of ILI residuals; filtered SARI diagnostics describe remaining serial dependence. Low SARI P values indicate residual serial dependence and make the white-noise limits approximate.

Table S11. Stationarity diagnostics for positivity levels and first differences

Pathogen	Series	ADF level P	KPSS level P	ADF Δ P	KPSS Δ P
SARS-CoV-2	ILI	0.298	0.027	<0.001	0.100
SARS-CoV-2	SARI	0.160	0.036	0.016	0.100
Influenza virus	ILI	0.335	0.100	<0.001	0.100
Influenza virus	SARI	0.070	0.100	0.001	0.100
RSV	ILI	0.492	0.010	<0.001	0.100
RSV	SARI	0.742	0.010	<0.001	0.100
Adenovirus	ILI	0.096	0.010	<0.001	0.100
Adenovirus	SARI	0.008	0.030	<0.001	0.100
hMPV	ILI	0.872	0.010	<0.001	0.100
hMPV	SARI	0.859	0.010	<0.001	0.100
Parainfluenza virus	ILI	0.310	0.100	0.001	0.100
Parainfluenza virus	SARI	0.888	0.100	<0.001	0.100
Common human coronavirus	ILI	0.039	0.078	<0.001	0.100
Common human coronavirus	SARI	0.077	0.052	<0.001	0.100
Bocavirus	ILI	0.563	0.069	<0.001	0.100
Bocavirus	SARI	0.676	0.047	<0.001	0.100
Rhinovirus	ILI	0.104	0.100	<0.001	0.100
Rhinovirus	SARI	0.068	0.100	<0.001	0.100
Enterovirus	ILI	0.034	0.100	<0.001	0.100
Enterovirus	SARI	0.617	0.100	<0.001	0.100
M. pneumoniae	ILI	0.004	0.010	0.004	0.010
M. pneumoniae	SARI	<0.001	0.010	<0.001	0.010

ADF tests used an intercept and linear trend for levels and an intercept for first differences; the null hypothesis is a unit root. KPSS used the corresponding trend or level-stationarity null. Reported KPSS P values of 0.010 or 0.100 are table bounds. ADF rejected a unit root in all differenced series; KPSS did not reject stationarity in 20 of 22 differenced series.

Table S12a. Sensitivity to the maximum ILI lag window

Pathogen	Best lag AICc	Best lag BIC	q: lags 1-2	q: lags 1-4	q: lags 1-6	q: lags 1-8
SARS-CoV-2	3	3	0.049	0.031	0.014	<0.001
Influenza virus	7	7	<0.001	<0.001	<0.001	<0.001
RSV	3	2	<0.001	<0.001	<0.001	<0.001
Adenovirus	1	1	0.020	0.039	0.017	0.059
hMPV	3	2	<0.001	<0.001	<0.001	<0.001
Parainfluenza virus	1	1	<0.001	<0.001	<0.001	<0.001
Common human coronavirus	4	4	<0.001	<0.001	<0.001	<0.001
Bocavirus	1	1	0.802	0.925	0.938	0.651
Rhinovirus	6	2	<0.001	<0.001	<0.001	<0.001
Enterovirus	2	1	0.013	0.031	0.002	0.011
M. pneumoniae	3	1	0.017	0.039	0.102	0.062

AICc and BIC compared maximum ILI lag windows 1-8 with AR(4) outcome history and one annual Fourier harmonic. Each q column is a separate exploratory family of 11 pathogen-level omnibus tests.

Table S12b. Sensitivity to level and alternative outcome-history specifications

Pathogen	Levels q	AR(8) q	AR(8) LB P	AICc AR order	Selected-AR q	Selected-AR LB P
SARS-CoV-2	0.119	0.023	0.126	1	<0.001	0.581
Influenza virus	<0.001	<0.001	0.020	7	<0.001	0.048
RSV	<0.001	<0.001	0.818	1	<0.001	0.544
Adenovirus	0.008	0.117	0.189	2	0.095	0.277
hMPV	<0.001	<0.001	0.056	1	0.004	0.681
Parainfluenza virus	<0.001	0.010	0.075	2	0.003	0.041
Common human coronavirus	<0.001	<0.001	0.339	4	<0.001	0.133
Bocavirus	0.796	0.938	0.300	2	0.865	0.674
Rhinovirus	<0.001	<0.001	0.055	2	<0.001	0.179
Enterovirus	0.007	0.487	0.229	3	0.011	0.040
M. pneumoniae	0.880	0.204	0.044	2	0.004	0.805

Levels models used SARI and ILI positivity rather than first differences. AR(8) and AICc-selected AR models retained ILI lags 1-4 and one Fourier harmonic. Primary AR(4) Ljung-Box P values used lag 8 with model_df=4. AR(8) P values used lag 12 with model_df=8. For AICc-selected AR(p) models, the diagnostic lag was max(8, p + 4) with model_df=p. Thus, each dynamic specification retained at least four effective diagnostic degrees of freedom. HAC covariance was retained in every specification.

Table S13. Restricted recursive moving-block bootstrap sensitivity for the primary forward lag block

Pathogen	Observed F	Asymptotic q	Bootstrap P L=4	Bootstrap q L=4	Bootstrap P L=8	Bootstrap q L=8
SARS-CoV-2	3.015	0.031	0.133	0.162	0.115	0.147
Influenza virus	22.859	<0.001	<0.001	0.003	<0.001	0.003
RSV	5.933	<0.001	0.010	0.028	0.011	0.030
Adenovirus	2.778	0.039	0.129	0.162	0.117	0.147
hMPV	5.958	<0.001	0.018	0.032	0.025	0.045
Parainfluenza virus	5.599	<0.001	0.017	0.032	0.017	0.037
Common human coronavirus	12.155	<0.001	0.002	0.008	0.001	0.007
Bocavirus	0.222	0.925	0.954	0.954	0.958	0.958
Rhinovirus	8.654	<0.001	0.006	0.023	0.004	0.016
Enterovirus	3.122	0.031	0.103	0.162	0.120	0.147
M. pneumoniae	2.726	0.039	0.313	0.345	0.347	0.382

Each analysis used 3999 successful restricted recursive circular moving-block replicates generated under the reduced AR(4)-Fourier model. Reduced-model residuals were mean-centered and resampled in circular blocks. The observed ILI series was held fixed; the observed initial SARI level and first four SARI differences supplied the initial conditions, after which SARI differences were generated recursively and the full model was refitted. Bootstrap P values used (1 + the number of replicate HAC-Wald F statistics at least as large as observed)/(1 + the number of successful replicates). Inference is conditional on the observed ILI series. Bootstrap q values were adjusted across 11 pathogens separately for each block length. The reproducible implementation is included in Supplementary File S1.

Table S14a. Direct 2- and 4-week forecast comparisons based on mean squared error

Pathogen	Horizon	Origins	MSE gain	95% CI	Raw P	q
SARS-CoV-2	2	37	-0.156	-0.549 to 0.095	0.400	0.526
Influenza virus	2	37	1.021	0.145 to 2.070	0.036	0.401
RSV	2	37	0.048	-0.311 to 0.400	0.760	0.760
Adenovirus	2	37	-0.011	-0.039 to 0.015	0.397	0.526
hMPV	2	37	-0.082	-0.288 to 0.110	0.431	0.526
Parainfluenza virus	2	37	-0.030	-0.100 to 0.033	0.377	0.526
Common human coronavirus	2	37	-0.041	-0.110 to 0.029	0.247	0.526
Bocavirus	2	37	-0.014	-0.044 to 0.006	0.288	0.526
Rhinovirus	2	37	0.063	-0.124 to 0.275	0.540	0.595
Enterovirus	2	37	-0.012	-0.028 to 0.004	0.146	0.526
M. pneumoniae	2	37	-0.008	-0.025 to 0.008	0.325	0.526
SARS-CoV-2	4	35	-0.153	-0.509 to 0.097	0.374	0.374
Influenza virus	4	35	-0.500	-1.628 to 0.362	0.328	0.371
RSV	4	35	-0.159	-0.378 to 0.025	0.127	0.244
Adenovirus	4	35	-0.009	-0.019 to 0.001	0.102	0.244
hMPV	4	35	-0.121	-0.279 to 0.045	0.132	0.244
Parainfluenza virus	4	35	-0.154	-0.281 to -0.034	0.013	0.148
Common human coronavirus	4	35	-0.118	-0.282 to -0.002	0.093	0.244
Bocavirus	4	35	-0.025	-0.058 to 0.006	0.133	0.244
Rhinovirus	4	35	-0.110	-0.305 to 0.048	0.222	0.305
Enterovirus	4	35	-0.028	-0.087 to 0.025	0.338	0.371
M. pneumoniae	4	35	-0.010	-0.030 to 0.002	0.204	0.305

Positive gains favor the AR-harmonic plus ILI model. Direct models used the latest four SARI and ILI changes available at each forecast origin. Each longer-horizon comparison used 3999 paired circular moving-block bootstrap replicates with block length 4. The 95% percentile interval was calculated from the uncentered bootstrap distribution of mean gains, whereas the two-sided P value was calculated from the absolute centered-null distribution with plus-one correction. These finite-sample procedures are not algebraically equivalent; consequently, a percentile interval can narrowly exclude zero while the centered-null P value exceeds 0.05, as for the four-week common human coronavirus MSE gain. q values were adjusted across pathogens within each horizon.

Table S14b. Direct 2- and 4-week forecast comparisons based on mean absolute error

Pathogen	Horizon	Origins	MAE gain	95% CI	Raw P	q
SARS-CoV-2	2	37	-0.055	-0.171 to 0.030	0.266	0.643
Influenza virus	2	37	0.367	0.078 to 0.663	0.012	0.129
RSV	2	37	0.025	-0.094 to 0.157	0.695	0.695
Adenovirus	2	37	-0.010	-0.043 to 0.024	0.546	0.643
hMPV	2	37	-0.064	-0.189 to 0.048	0.306	0.643
Parainfluenza virus	2	37	-0.012	-0.055 to 0.029	0.584	0.643
Common human coronavirus	2	37	-0.044	-0.107 to 0.025	0.200	0.643
Bocavirus	2	37	-0.004	-0.019 to 0.010	0.547	0.643
Rhinovirus	2	37	0.027	-0.061 to 0.116	0.550	0.643
Enterovirus	2	37	-0.010	-0.036 to 0.015	0.407	0.643
M. pneumoniae	2	37	-0.009	-0.027 to 0.010	0.360	0.643
SARS-CoV-2	4	35	-0.043	-0.115 to 0.015	0.193	0.328
Influenza virus	4	35	-0.082	-0.300 to 0.134	0.451	0.551
RSV	4	35	-0.011	-0.091 to 0.069	0.795	0.795
Adenovirus	4	35	-0.010	-0.021 to 0.001	0.075	0.328
hMPV	4	35	-0.050	-0.115 to 0.017	0.133	0.328
Parainfluenza virus	4	35	-0.118	-0.183 to -0.052	<0.001	0.008
Common human coronavirus	4	35	-0.050	-0.116 to 0.005	0.108	0.328
Bocavirus	4	35	-0.026	-0.069 to 0.010	0.209	0.328
Rhinovirus	4	35	-0.027	-0.085 to 0.034	0.363	0.499
Enterovirus	4	35	-0.022	-0.089 to 0.039	0.502	0.552
M. pneumoniae	4	35	-0.009	-0.022 to 0.004	0.167	0.328

Positive gains favor the AR-harmonic plus ILI model. Confidence intervals and P values were calculated using the distinct percentile and centered-null bootstrap procedures described for Table S14a. Parainfluenza at four weeks had a negative FDR-significant MAE gain, indicating worse error for the augmented model; no pathogen showed an FDR-significant positive gain.

Table S15. Summary of cumulative coefficients across all rolling 72-week windows

Pathogen	Windows	Cumulative β range	Same sign (%)	FDR-significant windows (%)
SARS-CoV-2	24	0.500 to 0.931	100.0	83.3
Influenza virus	24	0.758 to 1.262	100.0	100.0
RSV	24	1.447 to 1.802	100.0	100.0
Adenovirus	24	0.245 to 1.004	100.0	29.2
hMPV	24	0.661 to 1.561	100.0	100.0
Parainfluenza virus	24	0.601 to 1.009	100.0	100.0
Common human coronavirus	24	1.646 to 1.797	100.0	100.0
Bocavirus	24	0.253 to 0.927	100.0	0.0
Rhinovirus	24	0.652 to 1.353	100.0	100.0
Enterovirus	24	0.291 to 0.521	100.0	29.2
M. pneumoniae	24	0.163 to 2.167	100.0	16.7

The 24 windows share 71 of 72 weeks with adjacent windows and are not independent validation samples. Within each window, joint-test P values were adjusted across 11 pathogens. The table describes temporal variation; Figure S3 displays coefficients and unadjusted 95% HAC confidence intervals.

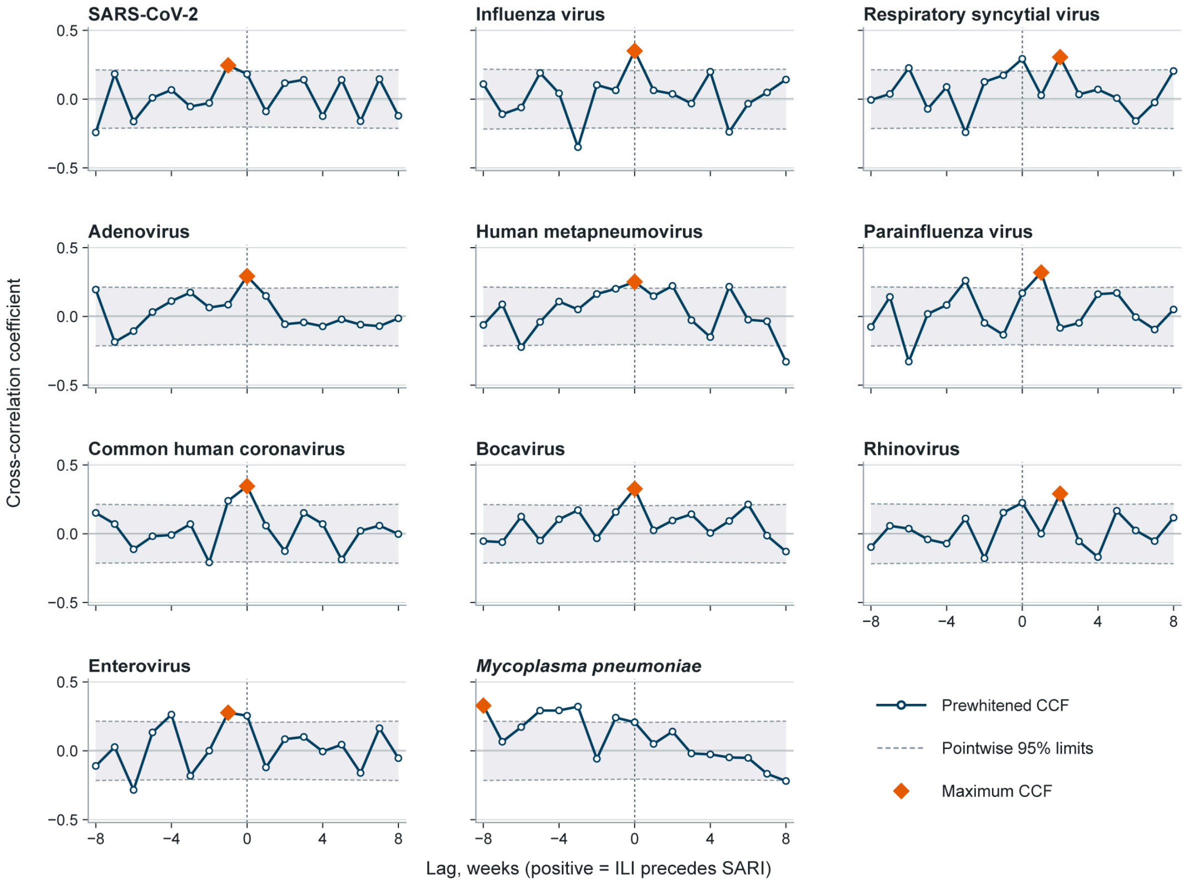

Figure S1. Prewhitened cross-correlations between ILI and SARI pathogen positivity. Curves show cross-correlation functions (CCFs) after pathogen-specific prewhitening of undifferenced positivity levels. Positive lags indicate that innovations in ILI preceded innovations in SARI. Dashed horizontal lines are approximate pointwise white-noise references for visual interpretation, not inferential thresholds. The CCFs are descriptive because eight filtered SARI series retained residual serial dependence. ILI, influenza-like illness; SARI, severe acute respiratory infection.

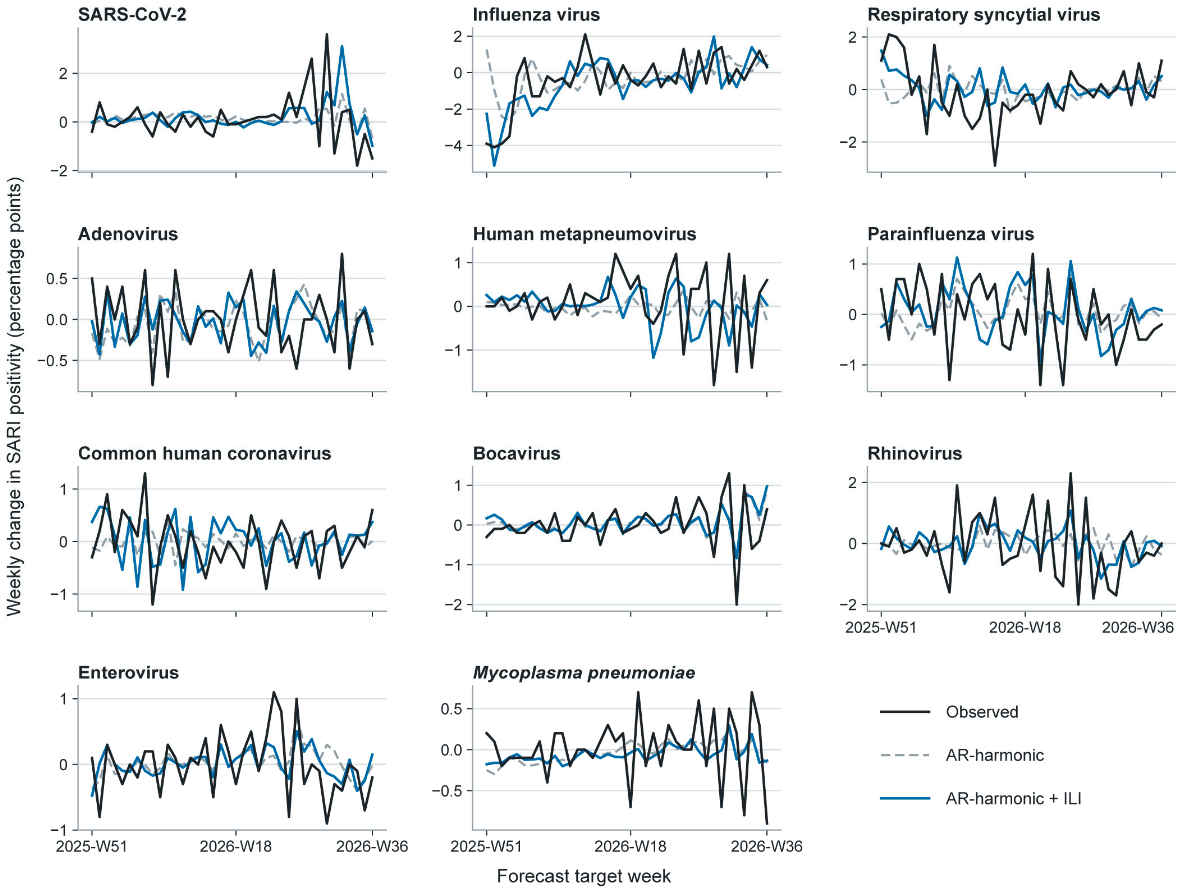

Figure S2. Observed and rolling-origin predicted weekly changes in SARI positivity. Black lines show observed weekly change in SARI positivity. Gray dashed lines show forecasts from the AR-harmonic model, and blue lines show forecasts from the AR-harmonic plus lagged ILI model. Each panel contains 38 one-week-ahead forecasts generated from an expanding training window. AR, autoregressive; ILI, influenza-like illness; SARI, severe acute respiratory infection.

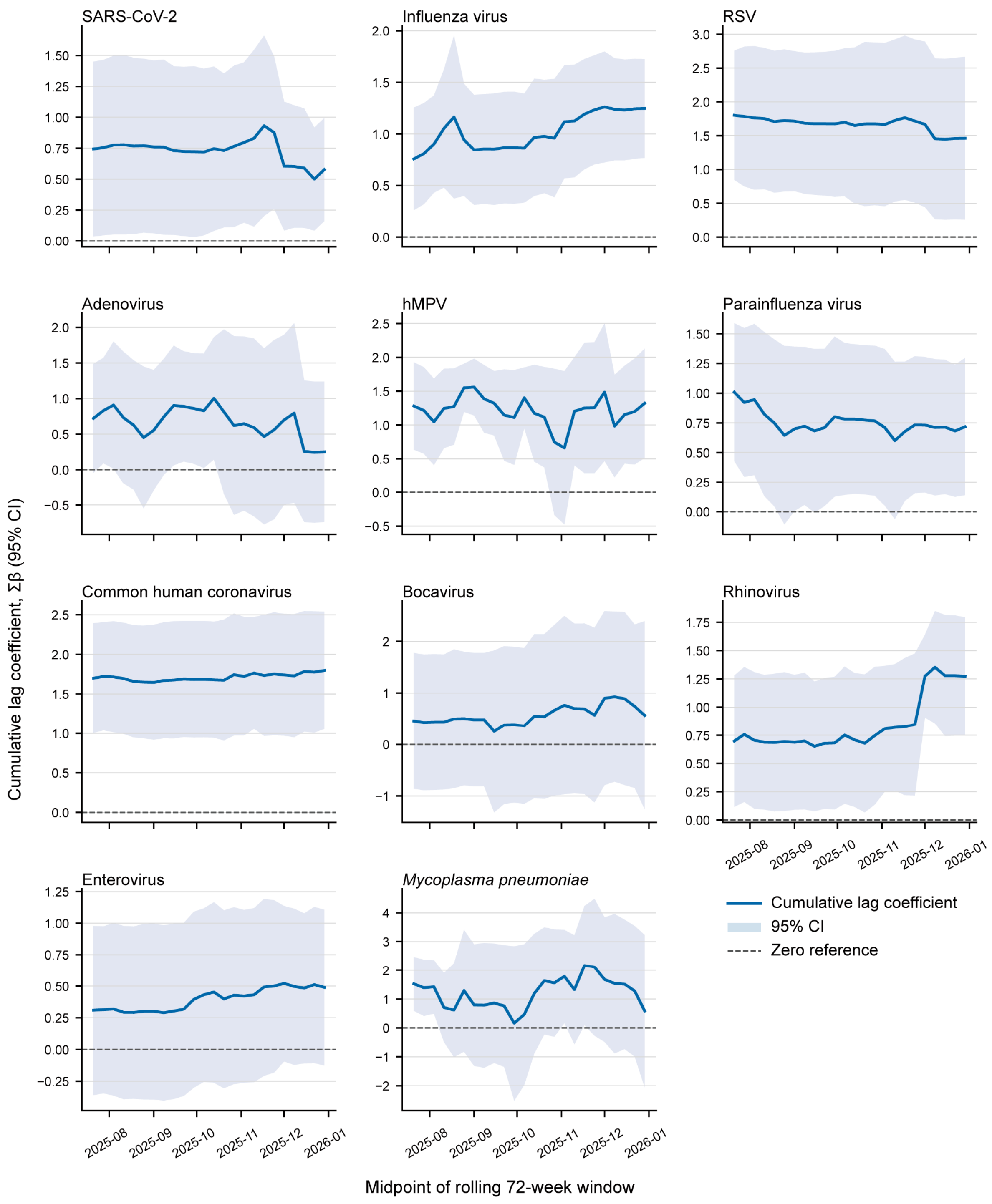

Figure S3. Rolling 72-week cumulative lag coefficients. Lines show the sum of ILI-change coefficients at lags 1-4 in each of 24 overlapping 72-week windows; shaded bands are unadjusted 95% HAC confidence intervals and dashed lines mark zero. Window midpoints are displayed on the x-axis. Adjacent windows share 71 weeks and are therefore descriptive of temporal variation rather than independent replications. ILI, influenza-like illness; HAC, heteroskedasticity- and autocorrelation-consistent.
